# Using adaptive designs for decision making within the optima trial: optimal personalized treatment of early breast cancer using multi-parameter tests

**DOI:** 10.1186/1745-6215-14-S1-O12

**Published:** 2013-11-29

**Authors:** Janet Dunn, Andrea Marshall, Amy Campbell, Nigel Stallard, Claire Hulme, Peter Hall, Helen Higgins, John Bartlett, Adrienne Morgan, Jenny Donovan, Andreas Makris, Luke Hughes-Davies, Rob Stein

**Affiliations:** 1 Warwick Medical School, University of Warwick, Coventry, UK; 2 Leeds Institute of Health Sciences, Leeds, UK; 3 Ontario Institute for Cancer Research, Toronto, Canada; 4 Independent Cancer Patients Voice, London, UK; 5 University of Bristol, Bristol, UK; 6 Mount Vernon Cancer Centre, Middlesex, UK; 7 Cambridge University Hospitals NHS Foundation Trust, Cambridge, UK; 8 University College London Hospitals NHS Foundation Trust, London, UK

## Background

OPTIMA has an adaptive design seeking to advance development of personalised medicine in breast cancer by assessing the value of multi-parameter tests, such as Oncotype DX, in a UK population of intermediate risk.

## Methods

OPTIMA prelim, the feasibility phase, aims to recruit 300 patients to evaluate performance and health-economics of a number of multi-parameter tests to identify test(s) to be used in the main trial and to establish the acceptability to patients and clinicians of randomisation. Patients are randomised to the standard arm or to the "test-directed treatment" arm according to the result of Oncotype DX test. The decision to roll forward into the main trial will be determined by the willingness of patients to be randomised, concordance and cost of the multi-parameter tests. Cost-effectiveness models will be based on the model developed in preparation for the OPTIMA trial, updated with contemporary evidence from the feasibility study and appropriate external data, e.g. the Ontario OncotypeDX field evaluation (prospective cohort study).

## Results

OPTIMA prelim opened in Sept 2012 and has 56 patients registered (46 randomised). TSC and DMEC agreed decision rules and encouraged external collaboration to provide additional confidence and power for any decisions.

## Conclusions

The success of OPTIMA relies on the integration of a multi-disciplinary team of methodologists, clinical experts and patients at all stages of the trial. The complexities of using adaptive design methodology and decision making to roll forward into the main trial are challenging but provide the most efficient use of patients and costs.

## Funding

This project was funded by the National Institute for Health Research Health Technology Assessment (NIHR HTA) Programme (project number 10/34/01). The views and opinions expressed therein are those of the authors and do not necessarily reflect those of the HTA programme, NIHR, NHS or the Department of Health.

